# Neural reactivation during human sleep

**DOI:** 10.1042/ETLS20230109

**Published:** 2023-12-06

**Authors:** Dan Denis, Scott A. Cairney

**Affiliations:** 1 Department of Psychology, University of York, York YO10 5DD, U.K.; 2 York Biomedical Research Institute, University of York, York YO10 5DD, U.K.

**Keywords:** cognition, learning and memory, sleep disorders

## Abstract

Sleep promotes memory consolidation: the process by which newly acquired memories are stabilised, strengthened, and integrated into long-term storage. Pioneering research in rodents has revealed that memory reactivation in sleep is a primary mechanism underpinning sleep's beneficial effect on memory. In this review, we consider evidence for memory reactivation processes occurring in human sleep. Converging lines of research support the view that memory reactivation occurs during human sleep, and is functionally relevant for consolidation. Electrophysiology studies have shown that memory reactivation is tightly coupled to the cardinal neural oscillations of non-rapid eye movement sleep, namely slow oscillation-spindle events. In addition, functional imaging studies have found that brain regions recruited during learning become reactivated during post-learning sleep. In sum, the current evidence paints a strong case for a mechanistic role of neural reactivation in promoting memory consolidation during human sleep.

## Introduction

After initial learning, newly formed memories undergo a period of *consolidation*, which broadly refers to the strengthening and stabilisation of memory traces over time. Sleep, a state where sensory input is diminished, appears to be conducive to memory consolidation processes [1–5]. Beyond passively protecting memory traces, however, recent work has indicated that sleep plays an *active* role in consolidation [6–8]. The neurophysiological mechanisms underpinning this memory function of sleep have thus been the focus of numerous empirical studies in recent years.

Memory reactivation, especially during non-rapid eye movement (NREM) sleep, has emerged as a leading candidate mechanism underpinning sleep's beneficial effect on memory [9]. Landmark studies in rodents have shown that patterns of neuronal firing observed at learning are replayed during sleep in temporally compressed sequences, primarily in hippocampus but also in coordination with learning-related cortical sites [10–14]. The phenomenon of replay has been linked to post-sleep behavioural performance. Not only are the amount of replay events correlated with pre- to post-sleep memory improvement [12], but techniques such as optogenetics have found that disrupting memory replay leads to a selective disruption of the consolidation of that memory [15–17].

**Figure 1.**
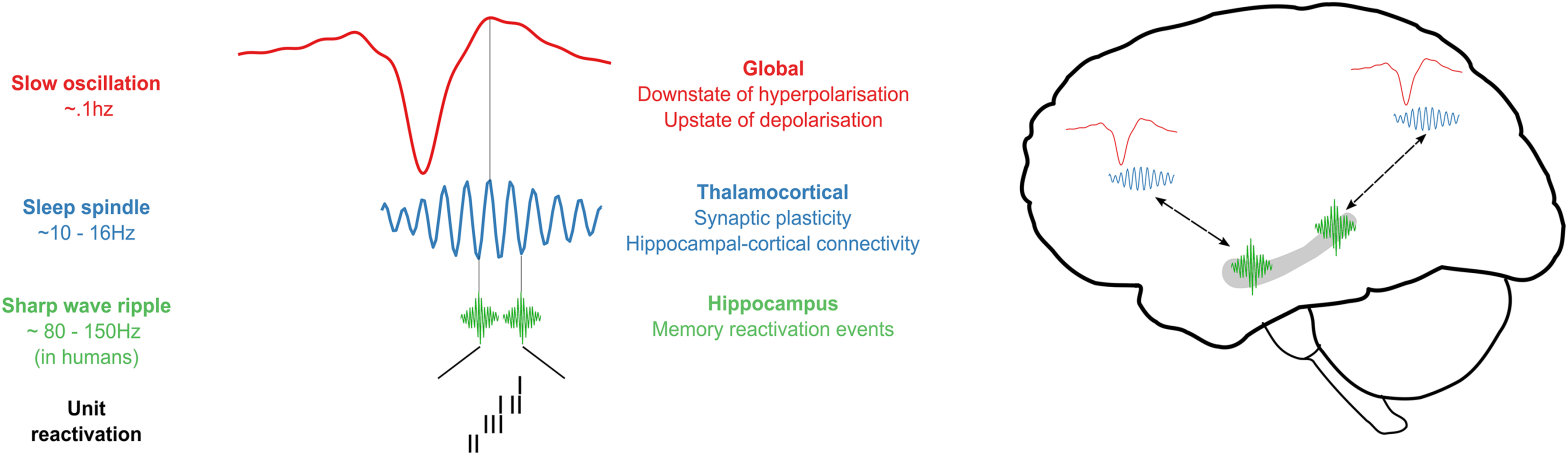
How neural oscillations support memory reactivation during sleep. Neural sequences coding for specific memories are reactivated during hippocampal sharp-wave ripples. Ripples nest into the excitable trough of sleep spindles: thalamocortical oscillations that facilitate information transfer between hippocampus and neocortex. Spindles in turn couple to the upstate of global slow oscillations, which reflect periods of brain-wide depolarisation, offering a critical time window for this hippocampal-cortical dialogue to occur.

The influential *Active Systems Consolidation (ASC)* framework posits that memory reactivation in hippocampus and neocortex during sleep supports the integration of newly acquired memories into long-term storage [18,19]. This hippocampal-neocortical dialogue is facilitated by a finely tuned interplay of three neural oscillations that characterise NREM sleep: high-frequency (∼200 Hz in rodents, ∼80–150 Hz in humans) hippocampal ripples, thalamocortical spindles (∼10–16 Hz), and global slow oscillations (SOs; ∼1 Hz) [9,20] (Figure 1). In hippocampus, ripples encompass reactivation events and are nested into the troughs of spindles, which in turn induce synaptic plasticity in learning-related circuits [21]. A subset of these spindle-ripple events are enwrapped in the excitable rising phase (upstate) of global SOs, which act as a pacemaker for information transfer between hippocampus and neocortex [22].

This hippocampal-cortical dialogue appears to follow a cortical-hippocampal-cortical loop in rodents, with the engagement of task-relevant cortical sites preceding associated reactivation in hippocampus [23–25]. It has been suggested that initiation by cortex ensures that the cortical sites are in a state that is conducive to plasticity [26]. Hippocampal-cortical connectivity is then sustained for a period following hippocampal reactivation, allowing for a spindle-mediated transfer of memory traces from hippocampus back to neocortex [26,27]. These findings emphasise the importance of not just the hippocampus in memory reactivation processes, but also the role that learning related-cortical sites play in initiating a cross-regional dialogue.

Rodent models have made a substantial contribution to our understanding of the neurophysiological mechanisms underpinning sleep-associated memory processing. This review aims to examine recent efforts to translate these findings into humans. We will first consider evidence for the coupling of ripples, spindles, and SOs during human NREM sleep. We will then turn our attention to evidence that the temporal coordination between these neural oscillations promotes memory reactivation and information transfer between hippocampus and neocortex. Finally, we will review evidence that specific brain regions that are involved in learning become reactivated during human sleep.

## Coupling of neural oscillations during human sleep

Traditional scalp electroencephalography (EEG) has been widely used to study coupling between cortical SOs and spindles. These studies have consistently shown that a subset of spindles preferentially couple to the rising phase of SOs [28–32]. Correlational studies have reported that the memory benefits of sleep are best when spindles consistently couple to the peaks of SOs [33–40]. Furthermore, these SO-coupled spindles are significantly better predictors of memory than uncoupled spindles [41–44], highlighting the importance of oscillatory coupling in memory consolidation. Moving beyond correlational evidence, electrical and acoustic stimulation has been used to enhance SO-spindle coupling [45,46], leading to improvements in memory [47,48].

Scalp EEG is limited by its spatial resolution, meaning that it is not possible to reliably detect signals coming from deep brain sources such as hippocampus. Therefore the triple-coupling of ripples, spindles and SOs (a key tenet of ASC) cannot be assessed. The only way to reliably detect human hippocampal ripples is via intracranial EEG (iEEG), where depth electrodes are directly implanted into hippocampus. This invasive procedure is only justified in clinical cases, such as assisting in the treatment of medication-resistant epilepsy. Studies in this patient group have confirmed the triple-coupling of ripples, spindles and SOs during human NREM sleep [49,50]. Furthermore, these coupled oscillations drive firing rates of individual hippocampal neurons [51], offering a putative mechanism through which neural oscillations facilitate the reactivation of newly formed memory traces in NREM sleep.

Oscillatory coupling has also been shown to facilitate information transfer during sleep, both within the medial temporal lobe [51] and between hippocampus and neocortex [27,52]. This cross-regional communication appears to be mediated by spindle activity that is locked to hippocampal ripples [27,52], suggesting that spindles serve to synchronise mnemonic processing across cortical and subcortical structures [27,53]. In line with rodent work, it appears that this spindle-ripple mediated, hippocampal-neocortical dialogue is initiated in neocortex, with connectivity sustained until after the ripple has terminated, allowing for the transfer of reactivated memory traces back to cortex [27,52]

A recent breakthrough study was able to causally link these oscillatory dynamics to the behavioural benefits of sleep for memory [54]. Using a deep brain stimulation protocol, the researchers timed the delivery of electrical stimulation in pre-frontal cortex to the excitable upstates of ongoing SOs. Compared with sham stimulation, electrical stimulation increased neuronal spiking during SO upstates and amplified temporal coupling between ripples, spindles, and SOs [54]. Remarkably, upstate stimulation also enhanced picture recognition accuracy the following day, as compared with the sham condition [54]. As an important control condition, the researchers stimulated during the SO downstate, a period of neuronal quiescence that should not be conducive to memory consolidation [9]. This is indeed what they found, with downstate stimulation not leading to any improvement in memory performance. Thus, not only does the triple coupling of ripples, spindles and SOs exist in humans, it directly promotes memory consolidation.

Both scalp and intracranial EEG have verified the presence of oscillatory coupling during human NREM sleep and have linked this to memory consolidation. We next turn our attention to research demonstrating that memory content is reactivated during human sleep.

## Electrophysiological evidence for memory reactivation during sleep

In human models, the reinstatement of neural activity that was present at learning is taken as evidence of memory reactivation [55]. As such, machine learning decoding approaches are often used to assess evidence of memory reactivation in the sleeping human brain [56–58]. In such analyses, neural signatures of learning are delineated during wakefulness and this information is used to determine if and when the same content emerges during sleep. For example, suppose EEG is monitored while an individual views images of objects and scenes; the machine learning algorithm can be trained on this EEG data to differentiate neural activity patterns unique to viewing objects and scenes. The machine learning training parameters (obtained from object and scene viewing) can then be applied to sleep EEG data obtained after learning to predict if and when object and/or scene memories are reactivated.

Although there is evidence that sleep-associated memory processing unfolds throughout the night [59], a major challenge for researchers is to determine where to look for memory reactivation. Two approaches have been adopted to address this issue. The first concerns *endogenous memory reactivation*, where researchers look for evidence of memory reactivation that emerges spontaneously. Analyses are focused around the time of ripple-spindle-SO events on the basis that this co-ordinated neural activity represents an electrophysiological correlate of offline memory processing. One challenge of investigating endogenous memory reactivation is that researchers have limited control over which memories are reactivated. This can be addressed with the second approach, known as *targeted memory reactivation* (TMR). With TMR, odours or sounds that are associated with newly learned materials prior to sleep are then re-presented to individuals during sleep in a bid to reactivate specific memories. Both approaches provide a precise timeframe for researchers to isolate evidence of mnemonic processing in sleep.

### Endogenous memory reactivation

Studies in rodents have documented reactivation of neuronal firing patterns in temporally compressed sequences during sleep. Extending this finding to humans, a patient case study recently reported evidence of memory reactivation during sleep at the level of single neurons in motor cortex [60]. It was shown that neural sequences associated with motor learning were re-expressed at a rate significantly above chance in sleep, and there was also evidence that memory reactivation was temporally compressed [60].

**Figure 2.**
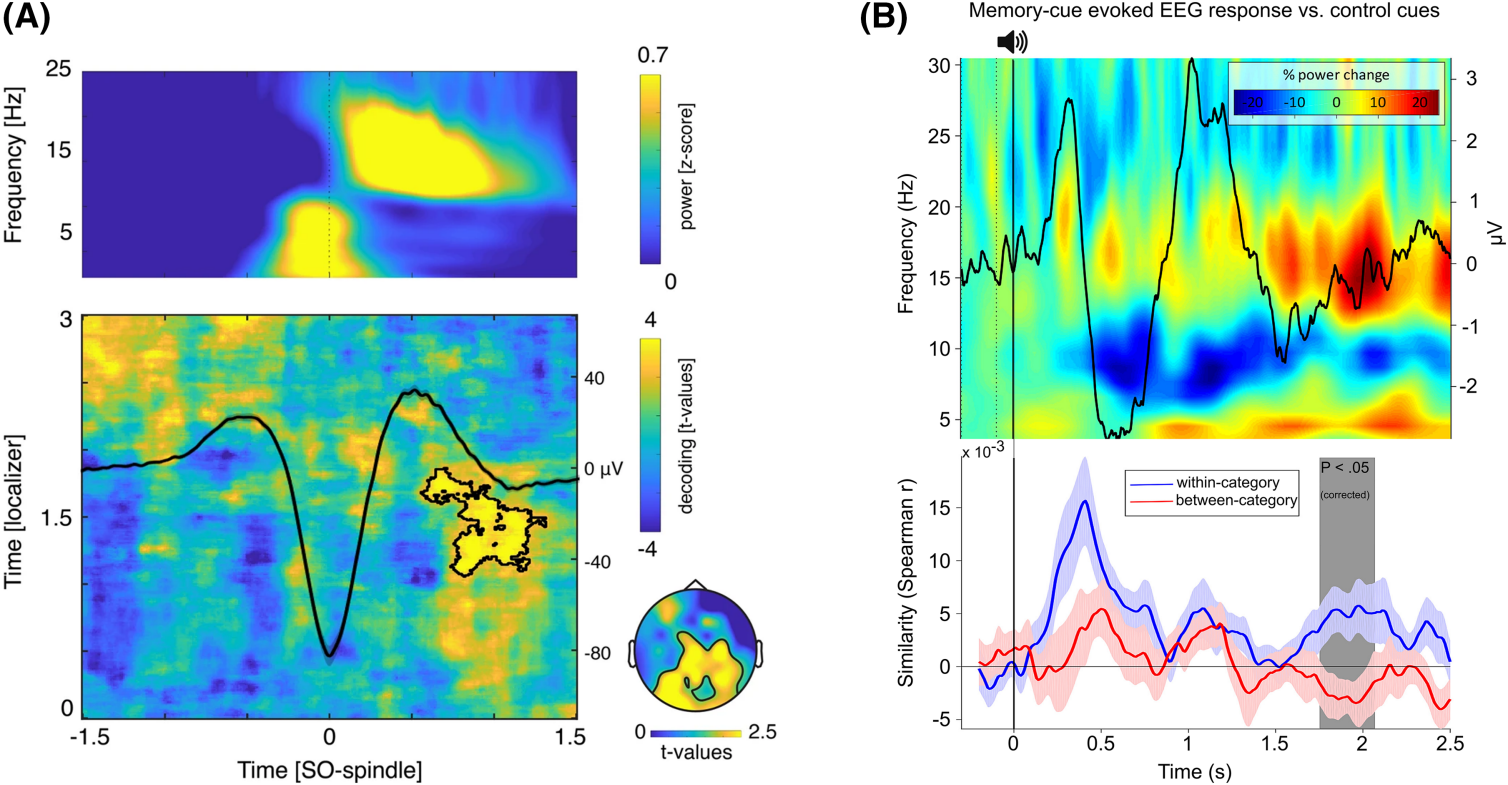
Electrophysiological evidence for memory reactivation in human sleep. (**A**) Endogenous memory reactivation. Top: Time frequency representation showing that spindle power (yellow blob at ∼15 Hz) is maximal around the peak of the SO (black line in bottom panel). Bottom: Machine-learning classification approaches can identify evidence of category-level memory reactivation during NREM sleep. Significant (i.e. above chance level) classification is highlighted with a black contour. Note the close alignment of significant classification to the SO peak [61]. (**B**) Targeted memory reactivation (TMR). Top: Following re-exposure to a memory cue in NREM sleep, there is a larger sleep spindle response than that emerging from previously unheard control cues, consistent with memory reactivation. Bottom: At the same time as this evoked spindle response, evidence of category-specific memory reactivation emerges in the EEG data [71]. All figure reproductions made under a Creative Commons Attribution 4.0 International License.

A recent scalp EEG study employed the machine learning decoding approach outlined above to test for memory reactivation during SO-spindle events. In line with the idea that SO-spindle coupling promotes memory reactivation, category-level features of previously encoded memories (objects or scenes) could be reliably discriminated near the peak of the ongoing SO (Figure 2A). Moreover, the magnitude of this category-level memory reactivation correlated with the precision of SO-spindle coupling and the benefits of sleep for memory retention [61]. Consistent with the putative function of coupled SO-spindle events in memory reactivation during sleep, evidence of category-level reactivation was not observed when the analyses were performed on either spindles or SOs in isolation.

Utilising iEEG, another study reported similar findings within hippocampus [62]. Here, the spontaneous re-emergence of wakeful learning patterns was detected throughout both quiet waking rest and NREM sleep. However, the behavioural benefits of sleep for memory were only correlated with reactivation that occurred during hippocampal ripples in NREM sleep. Together, these findings provide evidence of memory reactivation emerging spontaneously in the sleeping brain, and that reactivation supports overnight memory consolidation.

### Targeted memory reactivation

TMR biases memory reactivation towards memories that are associated with a sound or odour cue presented during sleep [63], leading to improved memory for cued relative to non-cued information [64]. TMR cues lead to a robust increase in sleep spindle activity, with the magnitude of this enhanced spindle response predicting the behavioural benefit of TMR [65–70]. This shows that TMR evokes patterns of neural oscillatory activity associated with memory reactivation and consolidation.

Recent studies have combined TMR protocols with machine learning methods to provide evidence of memory reactivation in sleep. This approach is based on the idea that, if a TMR cue elicits reactivation of a specific memory, then the evoked neural patterns should match the learning patterns for that memory, as compared with other memories associated with different cues. Using this approach, it has been demonstrated that categorical features (objects or scenes) of memories cued by TMR can be reliably decoded during the evoked spindle response [71] (Figure 2B). This timing is important, as it provides further evidence that sleep spindles are centrally involved in memory reinstatement processes. The magnitude of this effect also correlated with the behavioural benefit of TMR, thus linking memory reinstatement with behaviour. This basic finding, that memory content can be decoded following TMR cues, has been replicated several times across different domains of memory [72–75].

TMR cues are typically presented without consideration of what is occurring in the background EEG. However, as already discussed, memory reactivation appears to be optimal around the peak of the SO [61], and electrical stimulation applied during the SO upstate enhances memory, whereas stimulation in the downstate does not [54]. These findings would suggest that memory reactivation evoked by TMR cues should be optimal when TMR cues are presented during the SO upstate, as this is a critical time window for endogenous memory processing. Post-hoc analyses, which compare the behavioural benefit of TMR presented within different SO phases are equivocal on this point. One re-analysis found that only upstate TMR cues improved memory relative to no TMR, however the difference between upstate and downstate TMR was not statistically significant [76]. A different research group concluded that TMR cues presented during the SO downstate were more effective at improving memory compared with cues presented in the upstate [77]. A recent study experimentally manipulated the delivery of TMR cues to either the SO upstate or downstate, and found that neural reinstatement of memory content only occurred when cues were presented during the SO upstate [78]. While this finding is in line with theoretical predictions, it is clear that more work is needed to confirm the relationship between TMR effectiveness and ongoing neural oscillations

In sum, there is accumulating evidence from electrophysiological studies that memory reactivation occurs during human sleep, and this re-emergence of memory content is tightly coupled to SO-spindle-ripple events. In the next section, we turn to evidence from functional imaging methods, including functional magnetic resonance imaging (fMRI), which can more precisely detail reactivation in learning-related brain regions and networks.

## Functional imaging evidence for memory reactivation during sleep

Techniques such as fMRI or positron emission tomography (PET) offer unique insights into memory reactivation processes. First, it is possible to non-invasively measure hippocampal activity, which is not possible with scalp EEG. This is important, given the central role of the hippocampus in memory reactivation [9]. Second, rodent studies have emphasised the importance of learning-related cortical sites in both initiating reactivation sequences [24], and the subsequent expression of spindles at those cortical sites for inducing LTP and synaptic plasticity [21,79–82]. Compared with EEG, fMRI can more precisely measure *where* in the brain reactivation is occurring, allowing researchers to confirm whether learning-related sites are reactivated in human sleep.

In a landmark PET study, hippocampal activation observed during spatial navigation was observed again during post-learning sleep [83]. Hippocampal activity during sleep was higher than control groups who either performed no pre-sleep learning task, or engaged in non-hippocampal dependent learning, indicating that the observed hippocampal reactivation was selective for the spatial memories that recruited hippocampus during learning [83]. Many subsequent studies (primarily fMRI), have repeatedly shown that brain regions engaged at learning are re-engaged during subsequent sleep, with the level of re-engagement correlating with the behavioural benefits of sleep for memory consolidation [84–93].

**Figure 3.**
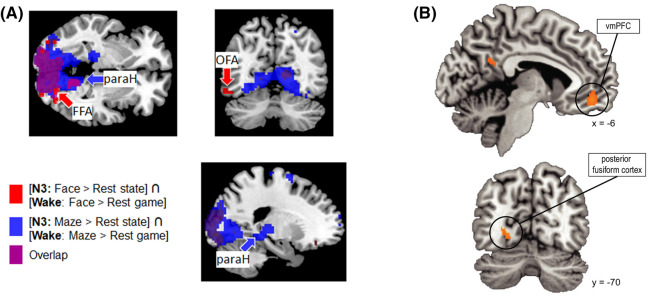
Functional imaging evidence for memory reactivation in human sleep. (**A**) Selective reactivation of learning-related brain regions during sleep. During the consolidation of a face task, there was selective reactivation of face-related brain areas, such as the fusiform face area. Conversely, during consolidation of a maze task, there was reactivation in brain areas associated with spatial navigation, such as parahippocampal cortex. Both tasks elicited reactivation in visual processing areas [89]. (**B**) Following the presentation of an odour-based TMR cue, category-level reactivation (i.e. discriminating between objects and scenes) was localised to pre-frontal and fusiform cortex regions, implicating these brain regions in the reactivation of category-level memory representations in sleep [88]. All figure reproductions made under a Creative Commons Attribution 4.0 International License.

Neuroimaging researchers have also documented reactivation of learning-related cortical regions during sleep. In one experiment where participants consolidated either a face or maze learning task, it was possible to discriminate which task was being consolidated based on reactivation within task-relevant regions [89] (Figure 3A). While both tasks led to reactivation in areas of visual cortex, consolidation of the face task uniquely led to reactivation in the fusiform face area (FFA). In contrast, the maze task promoted reactivation in parahippocampal regions.

A growing number of studies have utilised the power of simultaneous EEG-fMRI recordings to examine fMRI-derived neural reactivation that co-occur alongside sleep spindles detected in the concurrent EEG. This can address the question of whether memory reactivation emerges alongside the neural oscillations that are linked to memory reprocessing during sleep. In one such study, participants learned paired face-scene associates or performed a non-learning control task before sleeping in an MRI scanner with combined EEG recordings [85]. Results showed that activation in specific brain regions associated with processing of faces (FFA) and scenes (parahippocampal place area) re-emerged during sleep spindles. Similar findings have been reported for non-declarative procedural memories, whereby brain regions within a stratio-cerrobello-cortical network that are recruited during a motor sequence learning task are reactivated during post-learning sleep, also time-locked to sleep spindles [86,87].

TMR has also been combined with fMRI to elucidate memory reactivation during sleep. Using an odour-TMR paradigm, researchers paired each of four odours with four distinct categories (buildings, faces, animals, and tools). Two of the odours were re-represented during subsequent NREM sleep [88]. During odour re-presentation, it was possible to decode the memory category originally paired with the re-presented odour, implying category-level reactivation of the associated memories. Using the high spatial resolution offered by fMRI, they found reactivation occurred most strongly within the ventromedial pre-frontal cortex (vmPFC) and posterior fusiform cortex (Figure 3B). Importantly, category-level discrimination during wake was also localised to vmPFC, again highlighting the close spatial overlap between brain regions involved in learning and brain regions reactivated during sleep. The extent to which the odours reactivated the associated category was also predictive of memory recall performance after sleep, tying neural reactivation to the behavioural benefits of sleep for memory.

This finding of category-level reactivation during sleep is in agreement with findings observed in EEG studies [61,71]. It is important to highlight here how the EEG and fMRI results complement each other. The high temporal resolution and direct recording of neural oscillations available with EEG have demonstrated that memory reactivation is clocked by SO-spindle coupling events, in line with the primary predictions of ASC theory [9]. On the other hand, the high spatial precision of fMRI has confirmed rodent observations of memory reactivation in the learning-related cortical areas [24], pinpointing the brain regions that participate in reactivation with a level of specificity not possible with EEG.

## Future directions

Impressive progress has been made in detecting neural reactivation during human sleep. Complementary lines of evidence have confirmed that brain areas involved in memory encoding reactivate during subsequent sleep. Reactivation events occur most strongly during ripple-spindle-SO events, and numerous studies have linked the degree of reactivation to behavioural metrics of sleep-associated consolidation, implying a functional role of reactivation in enhancing memory. Despite this, several open questions remain.

First, work in rodents emphasises the temporally compressed sequential reactivation of learning-related cells in facilitating memory consolidation [10]. In humans, memory reactivation has been detected at broader spatial scales using MVPA techniques (e.g. [60,89]), but the temporal structure of the reactivation is still unknown. Sequential reactivation of single neurons in humans has been demonstrated to be feasible in principle [60], but future studies with multiple participants are needed to link such events to memory improvement. Future research could also consider employing tasks that have a sequential component to them (e.g. learning a sequence of pictures corresponding to different categories). Utilising a TMR protocol and MVPA analyses, it would be possible to examine sequential reactivation of the sequence following a TMR cue (e.g. if a learning sequence went object → scene → face, evidence for sequential reactivation would be obtained if an MVPA classifier trained on objects peaked first following the cue, followed by a classifier trained on scenes, then finally a classifier trained on faces). Evidence for time compression could also be evaluated by comparing the time courses of the reactivation during sleep with the activation patterns seen during wake, which would speak to the temporal compression seen in rodents.

Second, ASC places a heavy emphasis on oscillatory coupling in promoting memory consolidation. However, in humans at least, only a subset of spindles couple to SOs. The mnemonic function (if any) of uncoupled spindles has yet to be established, and more work is needed to understand the relative contributions of coupled and uncoupled spindles to offline memory processing. Relatedly, theoretical and empirical work has focused on the ‘fast' spindle subtype (∼13–15 Hz). So-called ‘slow' spindles (∼10–12 Hz) exhibit distinct topographies and SO-coupling profiles compared with fast spindles [28,30,41]. Although some work has shown that fast spindles are uniquely involved in memory consolidation [41,61], other research has indicated a mnemonic function of slow spindles [94]. Moreover, whereas scalp EEG is agnostic to the generator sources of detected sleep spindles, some iEEG work reported that slow spindles only emerge in cortex [49]. Fast spindles on the other hand occur in both cortical and subcortical structures, including the hippocampus. This preliminary work suggests that only fast sleep spindles exhibit the properties necessary to facilitate the cross-regional dialogue required by active systems consolidation theory. Therefore, the current ASC framework fails to consider the full dynamics of human sleep spindles, and future work is needed to refine our understanding of exactly which neural events best support memory consolidation.

With the growing evidence of memory reactivation in human sleep, a pertinent question is which memories are most likely to be reactivated. It is well known that long-term memory is biased in favour of personally or emotionally salient information [95–98], and some studies suggest that these kinds of memories are prioritised for consolidation during sleep [89,99] (though see [100]). Weakly encoded memories also appear to be reactivated preferentially during quiet wakefulness [101], receive the largest benefit from TMR in sleep [102,103], and are preferentially consolidated during SO-coupled spindles [41,104]. The mechanisms that underly selectively in consolidation are largely unknown. Based on the current ASC framework, it would be predicted that memory reactivation during SO-spindle events should be biased in favour of certain types of memories. This hypothesis could be tested using machine learning approaches to detect biased reactivation of highly salient information. By using a category-level learning paradigm (e.g. objects and scenes), researchers could tag the object category by informing participants that the retrieval of objects is associated with a high financial bonus. If this biases reactivation in favour of the high-reward category (objects in this example) a classifier should more frequently identify object-specific processing during SO-spindle complexes relative to scene-specific processing.

Relatedly, the hippocampal-cortical dialogue emphasised by ASC would suggest that hippocampal-dependent, associative memories would benefit the most from the mechanisms described by ASC. There is evidence that associative memories receive the largest behavioural benefit of sleep [105], and the degree of memory reactivation correlates with associative, but not recognition, memory performance [61]. These findings are not universal, however. Although recognition memory may benefit less from overnight consolidation, at least one study has linked memory reactivation in sleep to recognition memory performance [78]. Additionally, non-hippocampal procedural memories appear to be strengthened via the same oscillatory coupling mechanisms as declarative memories [42,43], suggesting that hippocampus is involved in memory consolidation even if it is not involved in encoding. Indeed, amnesic patients with hippocampal lesions can successfully learn a non-hippocampal task, but fail to consolidate it [106]. It appears therefore that multiple different memory systems are responsive to memory reactivation during sleep, but more research is required to better delineate potential differences between the consolidation of hippocampal and non-hippocampal forms of memory.

Finally, information transfer from hippocampus to neocortex during sleep is thought to facilitate the integration of related experiences into semantic knowledge, allowing us to draw on past experiences to make predictions about the future. As of yet, however, memory reactivation during human sleep has not been linked to such behavioural outcomes, with researchers primarily focusing on memory strengthening. Interestingly, category-level reactivation during sleep has been localised to vmPFC [88], a brain region which also acts as a hub for memory integration [107,108]. A recent study linked sleep spindles to the restructuring and integration of overlapping memory representations within vmPFC [108]. As such, one possibility is that category-level reactivation during sleep facilitates the integration of memories with shared categorical features, a process mediated by vmPFC. Such hypotheses should be explored in future work.

## Conclusions

The burgeoning literature on neural reactivation in human sleep has largely supported and translated findings from animal studies. Memory reactivation during sleep is facilitated by a finely tuned interplay of hippocampal ripples, thalamocortical spindles, and global SOs. This triple coupling supports a hippocampal-neocortical dialogue, allowing for the co-ordinated reactivation of memory traces in both hippocampus and neocortex, as well as facilitating information transfer in service of long-term storage. Future work should now start to address exciting questions regarding the potential selective nature of memory reactivation, and how memory reactivation sculpts long-term changes in neural memory representations.

## Summary

- Overwhelming evidence suggests that sleep benefits memory, leading to a strong interest in understanding the neural mechanisms underpinning this effect.
- Rodent studies have demonstrated memory reactivation to be a key mechanism underlying sleep's beneficial effect on memory, with reactivation occurring in tandem with cardinal non-rapid eye movement sleep oscillations.
- These findings have now started to be translated to humans. A growing number of studies have documented the re-emergence of memory content during sleep, with reactivation being closely linked to slow oscillation-spindle coupling events.
- Similarly, brain regions that are activated during the learning of new information are reactivated again during post-learning sleep, suggestive of learning-related memory reactivation.
- Future work now needs to better understand whether some memories are more likely to be reactivated than others, and what the long-term consequences of memory reactivation during sleep are on wakeful retrieval.

## Open Access

Open access for this article was enabled by the participation of University of York in an all-inclusive *Read & Publish* agreement with Portland Press and the Biochemical Society under a transformative agreement with JISC.

## Abbreviations

ASC: active systems consolidation theory
EEG: electroencephalography
FFA: fusiform face area
fMRI: functional magnetic resonance imaging
iEEG: intracranial electroencephalography
NREM: non-rapid eye movement sleep
PET: positron emission tomography
SO: slow oscillation
TMR: targeted memory reactivation
vmPFC: ventromedial pre-frontal cortex
